# Spatial patterns, ecological niches, and interspecific competition of avian brood parasites: inferring from a case study of Korea

**DOI:** 10.1002/ece3.1209

**Published:** 2014-09-05

**Authors:** Jin-Won Lee, Hee-Jin Noh, Yunkyoung Lee, Young-Soo Kwon, Chang-Hoe Kim, Jeong-Chil Yoo

**Affiliations:** 1 Department of Biology, Korea Institute of Ornithology, Kyung Hee UniversitySeoul, 130-701, Korea; 2 Bureau of Basic Ecological Research, National Institute of EcologySeocheon, 325-813, Korea; 3 Migratory Birds Centre, Korea National Park Research InstituteShinan, 535-917, Korea

**Keywords:** Avian brood parasitism, *Cuculus*, ecological niche, interspecific competition, spatial distribution, species distribution modeling

## Abstract

Since obligate avian brood parasites depend completely on the effort of other host species for rearing their progeny, the availability of hosts will be a critical resource for their life history. Circumstantial evidence suggests that intense competition for host species may exist not only within but also between species. So far, however, few studies have demonstrated whether the interspecific competition really occurs in the system of avian brood parasitism and how the nature of brood parasitism is related to their niche evolution. Using the occurrence data of five avian brood parasites from two sources of nationwide bird surveys in South Korea and publically available environmental/climatic data, we identified their distribution patterns and ecological niches, and applied species distribution modeling to infer the effect of interspecific competition on their spatial distribution. We found that the distribution patterns of five avian brood parasites could be characterized by altitude and climatic conditions, but overall their spatial ranges and ecological niches extensively overlapped with each other. We also found that the predicted distribution areas of each species were generally comparable to the realized distribution areas, and the numbers of individuals in areas where multiple species were predicted to coexist showed positive relationships among species. In conclusion, despite following different coevolutionary trajectories to adapt to their respect host species, five species of avian brood parasites breeding in South Korea occupied broadly similar ecological niches, implying that they tend to conserve ancestral preferences for ecological conditions. Furthermore, our results indicated that contrary to expectation interspecific competition for host availability between avian brood parasites seemed to be trivial, and thus, play little role in shaping their spatial distributions and ecological niches. Future studies, including the complete ranges of avian brood parasites and ecological niches of host species, will be worthwhile to further elucidate these issues.

## Introduction

Interspecific brood parasitism is a breeding strategy in which brood parasites lay their eggs in the nests of other species, called hosts, and shift parental duties onto them (Wyllie 1981; Davies 2000; Payne 2005). In birds, about 100 species (*ca*. 1% of all known bird species) are known to be obligate brood parasites; that is, they never build nests or provide food for their progeny (Davies 2000; Payne 2005). Because of the detrimental effect of brood parasitism, hosts often develop defensive strategies, such as parasitic egg discrimination and rejection, which leads to counter-adaptations such as egg mimicry by brood parasites (Brooke and Davies 1988; Davies and Brooke 1989; Stokke et al. 2002; Avilés et al. 2006; Kilner 2006). In addition, some host species develop further strategies such as egg color polymorphism, to defeat egg mimicry by brood parasites (Lee and Yoo 2004; Takasu 2005; Yang et al. 2010; Liang et al. 2012). These interesting aspects of avian brood parasitism have drawn much research interest, especially in the area of evolutionary biology as a model system of coevolution. Because of this, we have now accumulated much knowledge revealing their hidden life history (Rothstein 1990; Rothstein and Robinson 1998; Davies 2000).

Parasitic relationships, including brood parasitism, may also provide an excellent research subject in the field of macroecology for studying distribution patterns, ecological niches, and species interactions (Ricklefs 2010; Wisz et al. 2013), and this knowledge may in turn provide useful insights into not only evolutionary questions but also conservation issue. Currently, however, we have a relatively narrow range of information about this aspect of avian brood parasitism. As the availability of host species is a critical resource in the life history of avian brood parasites, the patterns of their distribution and abundance should be shaped not only by their own ecological needs related to climate and food availability but also by those of their host species (Davies 2000; Ducatez 2014). In particular, some avian brood parasites such as *Cuculus* species are known to have host specificity; in other words, each species or each individual within a species parasitizes a specific host species rather than exploiting multiple species, indicating that they may adopt different adaptive trajectories according to the host species on which they specialize (Marchetti et al. 1998; Gibbs et al. 2000; Nakamura et al. 2005; Davies et al. 2006; Madden and Davies 2006; Fossøy et al. 2011). This host specificity may provide a good opportunity to clarify fully the effect of biotic factors in the study of species distribution. Brood parasites may either adjust their range and ecological niche to those of any suitable hosts or confine their available host species to those in their current range and ecological niche. Such a tendency may vary among species or among individuals within a species, being a potential driving force of niche differentiation and speciation.

Different species of avian brood parasites may interact antagonistically to secure access to host species (Davies 2000). Competing species may end up separating their ranges, limiting their preference to a specific host species, or existing sympatrically but differentiating their ecological niches according to their host species. Furthermore, this competition over host species may underlie speciation in avian brood parasites (Davies 2000). Much circumstantial evidence supports the probability that such competition does occur in nature. For example, some host species in Japan and China are known to be parasitized as secondary hosts by multiple *Cuculus* species (Nakamura et al. 1998; Yang et al. 2012), implying the presence of potential competition between different brood parasites. Similar incidents were observed in Korea, where *Phoenicurus auroreus* seems to be used by two *Cuculus* species: *C. canorus* and *C. optatus* (J.-W. Lee, personal observation). Furthermore, when the competition between two species is released due to allopatric distributions, a species of brood parasites often exploits a typical host used exclusively by the other species where their ranges overlap, being an example of character release (Higuchi and Sato 1984). However, whether these incidences happened directly as a result of competition between species is still unclear, because the patterns of species distribution and interaction may also be influenced by many other factors such as abiotic environmental conditions (Dunson and Travis 1991; Lloyd and Palmer 1998; Martin 2001; Sexton et al. 2009). Further direct evidence of the presence of such competition and its outcome is still needed to clarify this issue.

In this study, we compared the spatial distribution patterns and ecological niches of five avian brood parasites breeding in Korea: *Cuculus canorus*, *C. optatus*, *C. micropterus*, *C. poliocephalus*, and *Hierococcyx hyperythrus*. They are known to not be globally threatened, although some of their local populations are reported to be declining (Birdlife International 2014). *C. canorus*, which is one of most studied avian brood parasites, has an extensive breeding range, occurring across Eurasia, and *C. optatus* is distributed from European Russia through Siberia and East Asia to the Pacific Coast of Eurasia during the breeding season (Birdlife International 2014). The three other species have much smaller breeding ranges, mainly restricted to Northeast Asia (Birdlife International 2014). In Korea, all five species are summer visitors (Lee et al. 2000) but information as to which species are regularly parasitized by each of the brood parasites is relatively limited because there are few relevant published studies. Nevertheless, some published as well as unpublished data are available, from which host species might be able to be inferred. *C. canorus* mainly use *Paradoxornis webbianus* in mainland Korea and *Emberiza cioides* in Jeju Island (Lee et al. 2000; Lee and Yoo 2004; Kang et al. 2009). Besides these species, *Phoenicurus auroeus*, *Saxicola torquatus*, and *Motacilla cinerea* are also observed to be occasionally parasitized by *C. canorus*. *C. optatus* seems to usually exploit *Phylloscopus coronatus* but occasionally *Phoenicurus auroeus*. In Jeju Island, it is also reported that *Terpsiphone atrocaudata* is parasitized by *C. optatus* (Kim 2011). The primary host of *C. poliocephalus* is known to be *Cettia diphone* and potentially *Troglodytes troglodytes* (Kang et al. 2009). Unfortunately, it is still unclear which species is regularly parasitized by *C. micropterus* in Korea. For *H. hyperythrus*, anecdotal observations, such as photographs of eggs or nestlings taken by local birdwatchers, suggest *Cyanoptila cyanomelana* as a potential primary host. Overall, each species of brood parasite is likely to exploit different primary host species, but, as mentioned earlier, it is also likely that some host species such as *Phoenicurus auroeus*, will be parasitized by multiple species of brood parasites.

The specific aims of this study are to identify the spatial and ecological niches of five sympatric brood parasites breeding in Korea and, based on this, to infer whether they actually compete with each other over spatial use and host availability. To achieve this, we first investigated their distribution patterns and relative abundances, and their ecological correlates. Secondly, we compared the ecological niches of each species estimated based on the climate conditions and altitude of the region where they were observed. Finally, species distribution modeling (SDM) using Maxent (Phillips et al. 2006) was carried out to determine ecologically important factors shaping their distribution patterns. The results from SDMs can also be applied to infer the presence of species competition by comparing predicted ranges with the realized distribution (Leathwick and Austin 2001; Guisan and Thuiller 2005; Elith and Leathwick 2009; Engler et al. 2013). Adopting this approach, we inferred the relative effect of ecological factors and species competition on the pattern of their spatial distribution.

## Materials and Methods

### Study area

**Figure 1:**
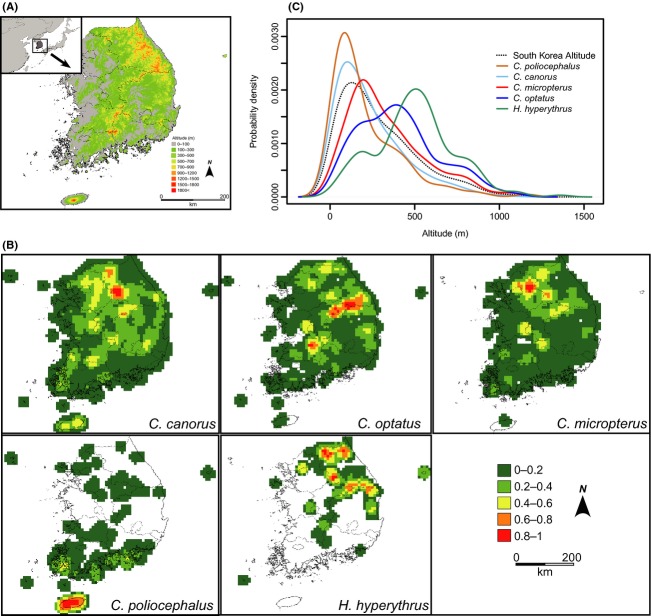
(A) The topographic map of South Korea and its largest island, Jeju-do. (B) Distribution map of the five species of avian brood parasites and the density of observed individuals analyzed with a 5-min cell size and circular neighborhood of 0.4 degrees. Note that the legends indicating the number of observed individuals are standardized for brevity and the maximum number of individuals represented by one vary among the species: 152 individuals for *C. canorus*, 52 for *C. optatus*, 111 for *C. micropterus*, 150 for *C. poliocephalus*, and 21 for *H. hyperythrus*. (C) Kernel density plots of the occurrence of the five avian brood parasites according to the altitude of South Korea.

The study area covers the mainland of South Korea and major islands, including the largest island, Jeju-do, which is located at the south of the Korean peninsula (Fig. 1A). South Korea is located in Northeast Asia and its approximate coordinates are 36°N, 128°E. About 70% of the mainland of South Korea consists of uplands or mountains, most of which are in mountain ranges along the eastern part of the mainland (Fig. 1A). The rest consists of lowlands lying along the west coast and major rivers. The highest elevation can be found at Jeju-do (1950 m). The climate of South Korea belongs to a temperate zone with four distinct seasons. Summers are humid and hot while winters are usually dry and cold. The weather in spring and autumn is mild but their durations are short.

### Study species and occurrence data

The georeferenced occurrence data of five avian brood parasites breeding in Korea were obtained from two sources of surveys: the third nationwide environmental study by the National Institute of Environmental Research and natural resource studies by the Korea National Park Research Institute. The former study was carried out from 2006 to 2012 across the whole of South Korea (*ca*. 99,000 km^2^), except for national parks (*ca*. 6581 km^2^), covering virtually all areas of South Korea. This survey was carried out based on 824 maps that cover South Korea at a 1:25,000 scale. Each map was divided into nine sections (3 × 3) approximately 4 × 5 km in size. Each year, 90–125 maps were surveyed during the survey period by over 70 trained ornithologists and birdwatchers. An actual survey was conducted at least once in each season in five sections near the center, excluding the four sections located at the four corners of the map. There were eight survey days per map. Both line transects and point counts were used as census methods, and the location and the number of birds seen or heard during the survey were recorded. The latter study, which covers the 20 national parks in Korea every year, was carried out by approximately 60 trained ornithologists and birdwatchers applying the same methods as the former survey. For each national park, we used 1 year of survey results chosen randomly from the data collected between 2006 and 2011 in order to be comparable to the former study in terms of the number of surveys and survey periods. The total number of survey days per year per national park ranged from 10 to 30, depending on the size of the park, but overall, similar survey efforts made. Combined, the studies of birds covered virtually all regions of mainland South Korea and major islands with similar amounts of survey effort. Furthermore, the males of each species produce loud, clear, and distinctive calls throughout their breeding seasons, which allowed us to easily spot the birds, even from a distance. Therefore, the quality of the occurrence data was unlikely to be biased in terms of the sampling efforts and survey area. We used the occurrence data collected between April and August when those avian brood parasites breed in Korea as this study was focused on breeding birds.

### Spatial and environmental analyses

The occurrence data were first plotted in DIVA-GIS 7.5 (Hijmans et al. 2012) to visually verify data precision, and then the species richness and distribution patterns of each species were analyzed based on point to grid analysis with a raster cell size of 0.0833 (*ca*. 7 km) and a circular neighborhood option of 0.4 degrees (*ca*. 35 km). Throughout the study, we used the same cell size and circular neighborhood option as above when we extracted the number of individuals recorded in each cell for density analysis. Altitude and land cover data were extracted from the SRTM 30 (available at: http://srtm.csi.cgiar.org) and GLC 2000 (available at: http://bioval.jrc.ec.europa.eu/products/glc2000/products.php) databases, respectively. Current climatic data within the study area were obtained from the most commonly referenced database, Worldclim v. 1.4 (Hijmans et al. 2005), with a resolution of 2.5 arc-min. Nineteen bioclimatic variables used in the Worldclim database are derived from monthly temperature and precipitation values, representing averages from 1950 to 2000 (Hijmans et al. 2005). Because some of those bioclimatic variables are highly intercorrelated, we carried out a principal component analysis (PCA) to generate an uncorrelated dataset, and finally, we adopted the first three principal components (PCs) with eigenvalues >1 after varimax rotation by the Kaiser criterion for the analysis of ecological niches. Visual comparison of the ecological niche and altitude that each species took up was carried out using kernel probability density plots in R version 3.0.2 (R Core Team 2013), and a Pianka index was calculated using ECOSIM 7.0 (Entsminger 2012) to quantify the degree of niche overlap between species. Values of this index close to 1.0 indicate a complete overlap of niches between species, while values close to 0 represent clear niche separation between species. The statistical significance of the observed overlap was tested using the randomization algorithm built into ECOSIM 7.0 (Entsminger 2012).

### Species distribution modeling

To predict the area of potential distribution, we carried out species distribution modeling using Maxent vers. 3.3.3k (Phillips et al. 2006). The 19 bioclimatic variables, altitude, and the type of land cover were included as environmental variables in the model. Despite potential over-parameterization due to high correlations between variables, we included all bioclimatic variables in order to explore all possibilities. This could be allowed in Maxent, because the program has a smoothing procedure called regularization, which relaxes the need to only choose uncorrelated environment variables (Phillips et al. 2006; Hastie et al. 2009; Elith et al. 2011). A randomly selected 75% of the presence records were used for model training and the remaining 25% for test points. We set subsample for the run-type option and 5000 for the maximum number of iterations of the optimization algorithm. For other options, we applied the default settings. The procedures were replicated 15 times, from which we obtained average values. For model evaluation, we provided the score of the area under the receiver operating characteristic curve (AUC). Although the AUC is one of the most widely used parameters to evaluate model performance, it is highly sensitive to model conditions such as the size of samples and backgrounds, especially in a model using presence-only data, such as Maxent (Townsend Peterson et al. 2007; Lobo et al. 2008; Phillips and Dudík 2008; Phillips et al. 2009; Jiménez-Valverde 2012). However, it is also true that there is no promising alternative to this for model evaluation. Therefore, we provided not only AUC scores but also training and test omission rates at 10% training presence (Jiménez-Valverde et al. 2008). The main environmental factors that considerably contributed to the Maxent model were determined by a jackknife test using AUC on test data. The logistic output of the model prediction for habitat suitability was projected in DIVA-GIS 7.0 (Hijmans et al. 2012), in which 0 represents non-suitable and 1 indicates fully suitable. We also generated a binary raster of potential distribution (presence/absence) using the logistic threshold of ten percentile training presence, from which we derived the predicted area of the sympatric presence of multiple species.

## Results

### Distribution patterns and species interactions

The number of data points used in the spatial analysis varied among species: 3462 for *C. canorus*, 1533 for *C. micropterus*, 858 for *C. optatus*, 526 for *C. poliocephalus*, and 191 for *H. hyperythrus*. These differences may reflect the relative abundances of the five species in Korea. They also showed various distribution patterns according to species. *C. canorus* was the most common, observed in almost all regions of South Korea, but with a higher density in the north (Fig. 1B). *C. micropterus* showed a similar distribution pattern to *C. canorus*. *C. optatus* and *H. hyperythrus* were less common than the former two species, mainly occurring along the mountain range, but the distribution of *H. hyperythrus* was much more restricted to higher elevations. The density of *C. poliocephalus* was highest in Jeju Island, followed by the southern part of mainland Korea. In mainland regions, they seemed to be much rarer and patchily distributed (Fig. 1B). Overall, the species were likely to have different altitudinal preferences (Fig. 1C). The lowest median elevation was found in *C. poliocephalus* (141 m; 1st–3rd quartiles: 67–319 m), and the highest was in *H. hyperythrus* (487 m, 1st–3rd quartiles: 369–602 m). In between them, the median elevations for *C. canorus*, *C. micropterus*, and *C. optatus* were 184 m (1st–3rd quartiles: 86–344 m), 271 m (1st–3rd quartiles: 176–455 m), and 381 m (1st–3rd quartiles: 369–602 m), respectively. Considering the frequency distribution of altitudes in South Korea, *C. canorus* and *C. poliocephalus* seemed to prefer lower altitudes while *C. micropterus*, *C. optatus*, and *H. hyperythrus* appeared to be observed more frequently at higher altitudes (Fig. 1C). The degree of altitudinal overlap estimated by the Pianka index was largest between *C. poliocephalus* and *C. canorus* (0.98) and smallest between *H. hyperythrus* and *C. poliocephalus* (0.48).

**Figure 2:**
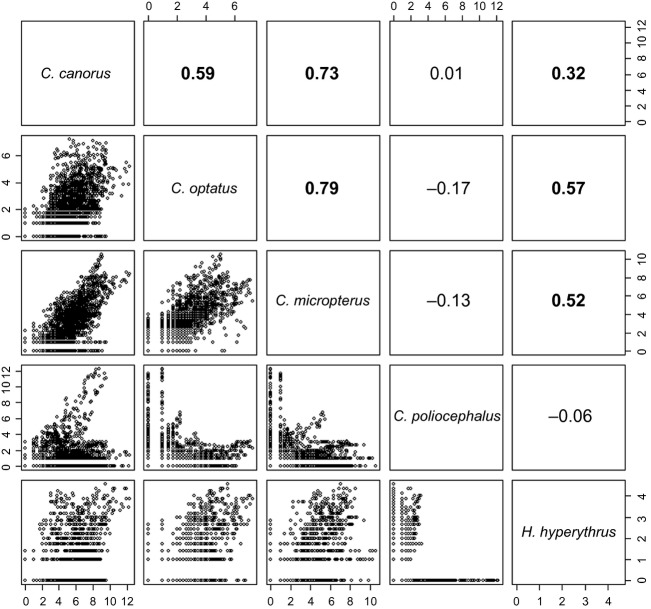
The pairwise comparison of the relative occurrence between the species. The dots in the lower diagonal represent the square-rooted number of individuals observed in 5-min cells with circular neighborhood of 0.4 degree. The numbers in the upper diagonal represent the spearman rank correlation coefficients of the counterparts. Statistically significant relationships are in bold.

The number of observed individuals of species occurring sympatrically appeared to be positively correlated with each other (Fig. 2). Among those, *C. optatus* and *C. micropterus* showed the strongest correlation (*r*_s_ = 0.79), and the next was between *C. canorus* and *C. micropterus* (*r*_s_ = 0.73). *C. canorus* and *C. optatus* also showed a positive correlation in abundance, but the strength was weaker (*r*_s_ = 0.59). Due to geographic segregation, the abundance of *C. poliocephalus* was not related to those of other species, but in the places where the abundance of *C. poliocephalus* was high (>10; e.g., Jeju island), its frequency of occurrence was positively correlated with that of *C. canorus* (*r*_s_ = 0.70). The abundance of *H*. *hyperythrus* was also positively correlated with those of *C*. *canorus*, *C. optatus*, and *C*. *micropterus*, but the relationship was moderate (Fig. 2).

### Realized ecological niche and its overlap

To test for differences in ecological niches between these five species, principal component analysis (PCA) was applied. The first three PCs with eigenvalues larger than one explained 91.6% of the total variance of the data (Table 1). PC1 best explained the variability of winter temperature and the seasonality of weather including temperature and precipitation. The winter temperature decreased with increasing PC1, while the seasonality of weather increased with increasing PC1. PC2 was most related to the variability of summer temperature and winter precipitation. The increasing PC2 represented lower summer temperature and heavier winter precipitation. PC3 mainly explained the variability of summer precipitation; the value of summer precipitation was positively correlated with PC3.

**Table 1:** The results of the principal component analysis of the 19 bioclimatic variables extracted from the areas of South Korea. The first three PCs with eigenvalue lager than one were represented here. The percentages in parentheses indicate the amount of variation explained by each PC, and the components that were loaded most highly for each parameter are in bold

Variables		PC1 (56.1%)	PC2 (23.4%)	PC3 (12.1%)
Bio1	Annual mean temperature	**−0.26**	−0.22	0.17
Bio2	Mean diurnal range	**0.26**	−0.13	−0.14
Bio3	Isothermality	0.14	−0.08	**−0.26**
Bio4	Temperature seasonality	**0.29**	−0.12	0.01
Bio5	Max temperature of warmest period	−0.10	**−0.41**	0.20
Bio6	Min temperature of coldest period	**−0.30**	−0.05	0.10
Bio7	Temperature annual range	**0.29**	−0.13	−0.03
Bio8	Mean temperature of wettest quarter	−0.16	**−0.37**	0.19
Bio9	Mean temperature of driest quarter	**−0.29**	−0.05	0.14
Bio10	Mean temperature of warmest quarter	−0.16	**−0.36**	0.24
Bio11	Mean temperature of coldest quarter	**−0.29**	−0.10	0.11
Bio12	Annual precipitation	0.01	0.32	**0.45**
Bio13	Precipitation of wettest period	0.25	0.05	**0.36**
Bio14	Precipitation of driest period	−0.20	**0.31**	−0.04
Bio15	Precipitation seasonality	**0.28**	−0.14	0.18
Bio16	Precipitation of wettest quarter	0.20	0.17	**0.43**
Bio17	Precipitation of driest quarter	−0.22	**0.30**	0.01
Bio18	Precipitation of warmest quarter	0.22	0.14	**0.40**
Bio19	Precipitation of coldest quarter	−0.22	**0.30**	0.02
Eigenvalue		10.7	4.5	2.3

Total explanation power 91.7%.

**Figure 3:**
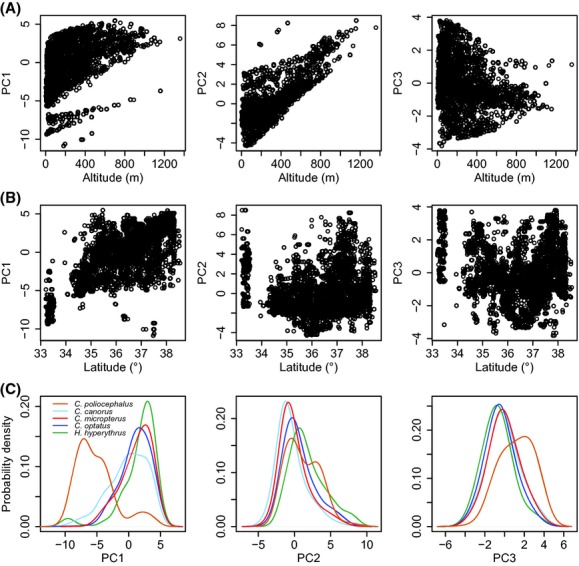
The patterns of association between the first three principal components and (A) altitude and (B) latitude. (C) The smoothed frequency distributions (kernel density plot) of the first three principal components that each species occupies.

These PCs were variously associated with altitude and latitude (Fig. 3A and B). PC1 was positively correlated with both latitude (*r*_s_ = 0.70) and altitude (*r*_s_ = 0.51), while PC2 was strongly associated with higher altitude (*r*_s_ = 0.72) but not with latitude (*r*_s_ = 0.08). PC3 appeared to be negatively correlated with altitude, but the effect was weak (*r*_s_ = −0.28), and no clear correlation was found with latitude (*r*_s_ = −0.04). These correlations explain well the characteristics of PCs in relation to altitude and latitude, indicating that PCs fully represent proper surrogates for the environmental components considered here and the interpretation of PCs is appropriate.

The estimation of the probability density for PC1 showed that *C. poliocephalus* had a differentiated range from the other four species (Pianka index: 0.39 for *C. canorus*, 0.23 for *C. micropterus* and *C. optatus*, and 0.17 for *H. hyperythrus*; Fig. 3C). Among those four species in which the substantial range of PC1 overlapped with each other (the Pianka index ranged from 0.84 between *C. canorus* and *H. hyperythrus* to 0.98 between *C. micropterus* and *C. optatus*), *C. canorus* had the broadest range of PC1 (Fig. 3C). There was a tendency for sequential variation in PC2, with the broadest range for *C. poliocephalus*, but the overall range of PC2 also widely overlapped among the five species (the Pianka index ranged from 0.66 between *C. canorus* and *H. hyperythrus* to 0.98 between *C. canorus* and *C. micropterus*; Fig. 3C). Similar to PC1, *C. poliocephalus* had a differentiated range in PC3 from the other species, but the extent was smaller (the Pianka index ranged from 0.63 between *C. poliocephalus* and *H. hyperythrus* to 0.99 between *C. canorus* and *C. micropterus*). Taken together, the overall ecological niches of the five species of avian brood parasites breeding in Korea overlapped significantly more than expected by chance (Table 2).

**Table 2:** The values of Pianka index (PI) showing the degree of overall niche overlap among the five species of avian brood parasites breeding in South Korea

	Mean of PI				Variance of PI		
Variables	Observed	Simulated^1^	ES^2^	*P*	Observed	Simulated	*P*
PC 1	0.66	0.50	3.17	<0.01	0.13	0.03	<0.0001
PC 2	0.86	0.48	6.96	<0.0001	0.01	0.03	0.03
PC 3	0.86	0.61	6.12	<0.0001	0.02	0.02	n.s.
Altitude	0.77	0.47	5.57	<0.0001	0.03	0.03	n.s.

1 The number of iteration for simulation is 10,000.

2 Standardized effect size: (Observed index-Simulated index)/(Standard deviation of simulated indices).

### Species distribution modeling

The average training and test AUC values for the Maxent models predicting habitat suitability for the five species of brood parasites ranged from 0.713 in *C. canorus* to 0.949 in *H. hyperythrus* and from 0.665 in *C. canorus* to 0.886 in *H. hyperythrus*, respectively (Table 3). The omission rates of test samples at 10% training sample presence also varied from 0.151 in *C. canorus* to 0.305 in *H. hyperythrus*. Overall, the model of *C. canorus* showed the smallest AUC value with the smallest test omission rate; in contrast, *H. hyperythrus* represented the largest values in AUC and test omission rate (Table 3).

**Table 3:** Summary of the Maxent models for the five species of avian brood parasites breeding in South Korea. Numbers in parentheses represent the number of samples

Species	Training AUC	Test AUC	Logistic threshold^1^	Training omission^1^	Test omission^1^
*Cuculus canorus*	0.713 (1268)	0.665 (422)	0.410	0.099	0.151
*Cuculus micropterus*	0.804 (608)	0.751 (202)	0.346	0.099	0.174
*Cuculus optatus*	0.821 (407)	0.764 (135)	0.356	0.098	0.182
*Cuculus poliocephalus*	0.925 (168)	0.879 (55)	0.160	0.095	0.181
*Hierococcyx hyperythrus*	0.949 (96)	0.886 (31)	0.361	0.094	0.305

AUC, operating characteristic curve; Number of background points are 8453.

1 Values at 10% training presence.

**Figure 4:**
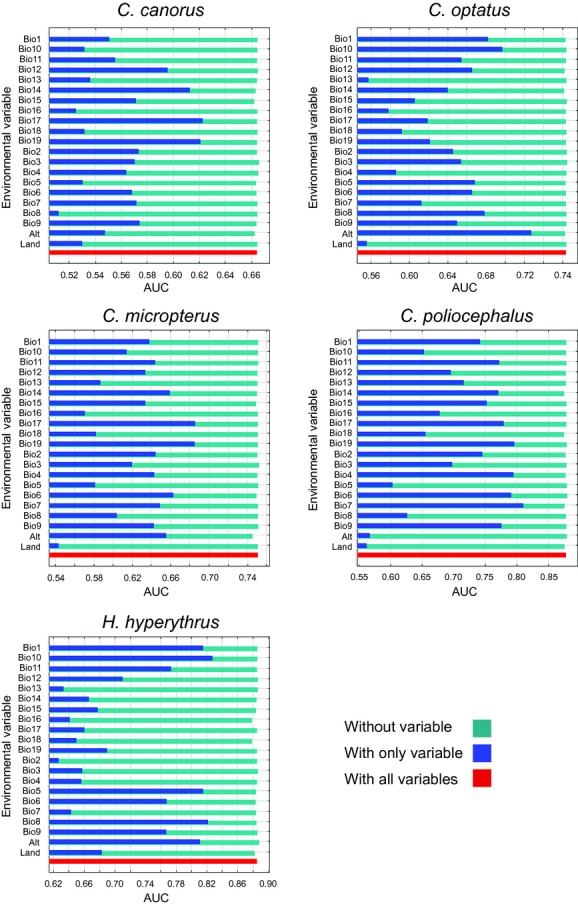
The results of the jackknife test of variable importance, using operating characteristic curve (AUC) on test data. The different colors of the bars indicate the value of AUC on test data when only a single variable is included in the Maxent model (blue) and when the variable is omitted from the model (green), respectively. The environmental variable that generates the highest AUC when used in isolation or that decreases the AUC the most when it is omitted could be considered the most useful information.

The main environmental factors that were evaluated using the jackknife test varied among species (Fig. 4). The variables related to winter precipitation (Bio14, Bio17, and Bio19) played an important role in the model prediction for *C. canorus* and *C. micropterus*. However, for *C. optatus* and *H. hyperythrus*, which were mainly observed in high mountains, altitude, and summer temperature (Alt, Bio5, Bio8, and Bio10) had large contributions to the model prediction, while the variables related to winter weather (Bio6 and Bio19) and the seasonality of temperature (Bio4 and Bio7) had large contributions to the model prediction for *C. poliocephalus*.

**Figure 5:**
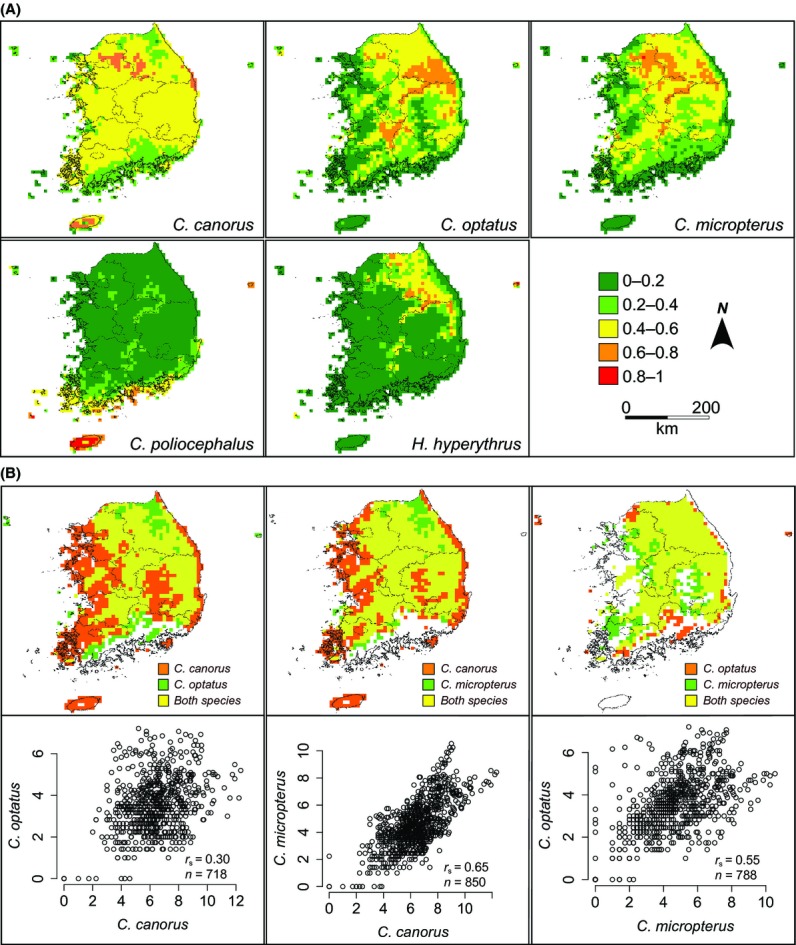
(A) Spatial prediction of the five species of avian brood parasites breeding in South Korea. Different colors represent the different degrees of occurrence probability (or habitat suitability). (B) The upper panel illustrates the overlap between predicted distributions of the three most widely distributed species in a pairwise manner. From the left panel, *C. canorus* versus *C. optatus*, *C. canorus* versus *C. micropterus,* and *C. optatus* versus *C. micropterus*, respectively. The logistic threshold of ten percentile training presence was applied to generate the presence/absence map. The orange and green colors indicate the areas where only one species was predicted to be presence while the areas with yellow color represent that both species are likely to occur together. The lower panel shows the association between the observed numbers of individuals (square-rooted) of focal species in the predicted areas (yellow) where both species occur sympatrically. The observed number of individuals was obtained from 5-min cells applying the option of circular neighborhood of 0.4 degrees.

The predicted distribution areas of suitable habitats for each species largely included the actual observed distribution areas of each species (Fig. 5A). *C. canorus* was predicted to be able to inhabit almost all areas of mainland Korea and Jeju Island, irrespective of altitude, with higher suitability for the northern part of mainland Korea. The majority of the mountain range was predicted to be the most suitable habitat for *C. optatus*, *C. micropterus*, and *H. hyperythrus*, but *H. hyperythrus* appeared to have a much narrower range, mostly restricted to high altitude areas. Meanwhile, Jeju Island was predicted to be an inappropriate habitat for those three species, especially for *C. micropterus* and *H. hyperythrus*. In contrast, Jeju Island was expected to be the most suitable habitat for *C. poliocephalus* (Fig. 5A). The southern part of Korea, mainly coastal areas including many islands, also appeared to be appropriate for *C. poliocephalus*, while in mainland Korea, suitable habitats were patchily distributed.

To identify the impact of antagonistic species interactions on the distribution of species, we generated rasters of presence/absence distribution for *C. canorus*, *C. optatus*, and *C. micropterus*, which had similar ecological niches, by applying the threshold rule of ten percentile training presence and then those binary rasters were overlaid in pairs to locate the areas where Maxent predicted that both focal species may sympatrically occur (Fig. 5B). Then, the number of individuals observed in each cell in those areas was compared, from which we found weak to moderate positive correlations between species in their relative abundance (Fig. 5B), indicating that species interactions between brood parasites may play only a minor role in shaping the pattern of their spatial distribution.

## Discussion

This study showed that the overall spatial ranges of five avian brood parasites breeding in Korea substantially overlapped with each other. Likewise, it appeared that they generally took up similar ecological niches, even though each species followed a different adaptive trajectory with respective host species. In addition, sympatric brood parasites showed positive relationships in their relative abundances, indicating that any antagonistic interaction between species may not be the first driving force shaping the pattern of the spatial distribution among avian brood parasites. Furthermore, combining these results with those of species distribution modeling confirmed again that their distribution ranges would be determined mainly by factors such as host distribution or climate conditions rather than species interactions among avian brood parasites.

Altitude is likely to be a major element characterizing the patterns of their distribution. Considering the frequency distribution of the altitude of Korea, *C. canorus* and *C. poliocephalus* were likely to be observed more frequently at lower altitudes. Alternatively, the data could also be interpreted to indicate that they tended to occur similarly across most of the range of altitudes without specific altitudinal preference, especially for *C. canorus*. In contrast, *C. optatus*, *C. micropterus*, and *H. hyperythrus* were observed more frequently at higher altitudes (>200 m) than expected by the frequency distribution of altitudes in Korea (Fig. 1C). In particular, the spatial range of *H. hyperythrus* was much more restricted to the higher parts of the mountain range (1st–3rd quartile: 369–602 m) than the other two species (1st–3rd quartile: *C. micropterus* 176–455 m; *C. optatus* 219–532 m). The biogeography of brood parasites should be closely coordinated with that of hosts. Thus, it is likely that these differences in altitudinal distribution may result from the altitudinal variation in the relative breeding density of their host species. For example, *Paradoxornis webbianus*, the primary host of *C. canorus* in mainland Korea, is a habitat generalist that breeds in diverse habitats at various altitudes from lowland reed beds to mountainous regions (Robson 2007). Alternatively, *C. canorus* may exploit different host species according to altitude because this species is known as a host generalist having many host-specific races compared to other study species. *Cettia diphone*, the primary host of *C. poliocephalus*, is also observed across various altitudinal ranges (Bairlein et al. 2006) and mainly inhabit the southern part of Korea, especially Jeju island with highest density (Lee et al. 2000), which may be one potential reason shaping the overall distribution pattern of *C. poliocephalus* in Korea (Fig. 1). In contrast, the potential host species of the other three species, such as *Phylloscopus coronatus* and *Cyanoptila cyanomelana*, are known to breed mainly in mountain forests (Bairlein et al. 2006; Taylor and Clement 2006). Ecological conditions such as climate and habitat types may also play a certain role in determining the altitudinal preferences of species. For example, the SDM for *H. hyperythrus* predicted that its range would be mainly restricted to mountain ranges with cool summer temperatures (Figs. 4, 5A), and the realized range was comparable (Fig. 1B). However, its primary host, *Cyanoptila cyanomelana*, may have a much broader altitudinal range than that of *H. hyperythrus* (Taylor and Clement 2006), indicating that host availability may not be the only factor determining the distribution of avian brood parasites. Negative species interactions between brood parasites could be an alternative factor limiting the altitudinal distribution of species, but the fact that the altitudinal ranges of all of the species substantially overlapped may diminish this probability. Anthropogenic development may be another important factor affecting not only altitudinal preference but also overall distribution pattern of species because higher altitude areas are generally less developed and thus have more natural habitats than lower areas in Korea. However, this may not be able to explain altitudinal variation of the distribution especially shown by three species (*C. optatus, C. micropterus, H. hyperythrus*), and we found that the effect of land cover types was small in the model (Fig. 4). Therefore, anthropogenic disturbance may affect the overall distribution pattern of species such as highest density in northeastern Korea but may not determine the altitudinal preference. Consequently, the altitudinal distribution of brood parasites seems to be closely related to altitudinal differences in ecological conditions and the distribution of their respective hosts.

In a relationship between brood parasites and hosts, niche conservatism, a tendency to retain ancestral ecological traits (Peterson et al. 1999; Wiens et al. 2010), may constrain the range of potentially available host species for brood parasites. On the other hand, antagonistic interactions between species of brood parasites may result in ecological niche differentiation between them. As with the pattern of spatial distribution, however, we found that the ecological niches of the five brood parasites were fairly similar to each other (Table 2). This implies that these closely related species tend to share ecological traits inherited from their common ancestor, even though they evolved along independent pathways of coevolution with their respective host species. In addition, contrary to expectations, our comprehensive analysis combining SDM results with observation data showed that interspecific competition between brood parasites over spatial use or host species may be trivial. However, our analysis was carried out using data extracted from a part of the entire range of the respective species. Therefore, further studies covering the additional or complete ranges of species would be worthwhile to further clarify this issue.

In SDMs, the AUC values have been widely but often uncritically used to evaluate model performance. However, it has been argued that the reliance of model evaluation on the AUC is questionable, especially when the modeling is based on presence-only data (Lobo et al. 2008; Jiménez-Valverde 2012). The AUC value is likely to be sensitive to the spatial extent of the background where pseudo-absence points are extracted; that is, increasing the area of the background may result in a larger AUC value, and vice versa. Similarly, the AUC is also influenced by the extent of distributional ranges of focal species, so the models for species with patchy distributions often generate larger AUC values than those of broadly distributed species, irrespective of sample size and actual model performance (Luoto et al. 2005; Elith et al. 2006; Hernandez et al. 2006; McPherson and Jetz 2007). In this study, the smallest training AUC value was gained from *C. canorus* (0.713) while the largest was from *H. hyperythrus* (0.949; Table 3). According to the model evaluation based solely on the AUC value, the model prediction for *H. hyperythrus* should be more accurate than that for *C. canorus*. However, the test omission rate at a 10% training presence of *H. hyperythrus* (0.305) was considerably larger than that of *C. canorus* (0.151), questioning the reliability of the AUC-based model evaluation (Table 3). Instead, we argue that the interspecific variation in the AUC values shown in this study seems to be derived from the difference in the evenness of the distribution; *C. canorus* was most widely distributed, while *H. hyperythrus* had the patchiest distribution (Fig. 1B).

In conclusion, the spatial patterns and ecological niches of avian brood parasites breeding in Korea appeared to be similar, despite potential conflicts over host availability. The results of this study imply that, contrary to expectation, interspecific competition between brood parasites may not be a primary driving force that differentiates the type of host species between brood parasites and that would ultimately lead to speciation. Alternatively, these could be achieved via somewhat indirect pathways such as host response to brood parasitism. In this study, we did not directly quantify the effect of the spatial distributions and ecological niches of host species on those of their brood parasites. Future studies including this approach will further clarify our understanding of the spatial dynamics and niche evolution in the system of avian brood parasitism. Furthermore, this aspect should be considered to be an important biotic factor in the study of future distributions according to climate change because the changing patterns of host distribution and host species composition may directly influence the spatial dynamics of brood parasites, showing the importance of biotic factors in the study of climate change, where their effects on species distribution have often been regarded as trivial.
